# The Doctrine of Legal Responsibility in Reference to Doubtful or Disordered States of the Mind, Practically Elucidated for the Use of Physicians and Jurists

**Published:** 1842-10

**Authors:** 


ART. IV.—*Die Lehre von der Zurechnungsfähigkeit bei Zweifelhaften Gemüthszuständen für Aerzte und Juristen praktisch dargestellt* Von Dr. ADOLPH SCHNITZER.—*Berlin*, 1840, pp. 372.

*The Doctrine of Legal Responsibility in reference to doubtful or disordered States of the Mind, practically elucidated for the use of physicians and jurists.* By Dr. ADOLPH SCHNITZER.—*Berlin*, 1840.

THE press of Germany has of late years yielded many works similar to that of which we are here about to give a brief notice. In our Thirteenth Number (Jan. 1839, p. 129) we had occasion to review a treatise by Professor Jörg, of Leipsic, on Legal Responsibility as it applies to females in the pregnant and parturient states. Dr. Schnitzer's work is of a more extended character: it includes not merely these conditions, but all those disordered states of the mental faculties which are usually comprised under the head of Insanity.

We find but little that is practical before reaching the third chapter. Here the author enters fully into the characters of puberty in the two sexes, with the morbid psychological changes which often accompany the development of the sexual organs. The fourth chapter is entirely devoted to the subject of pyromania, as it has been called by Marc and others. In Dr. Schnitzer's view, young persons between the ages of ten and twenty-four years, are especially liable to that disordered condition of mind which leads to acts of incendiarism. We do not, however, find that his remarks possess any originality: a great part of the chapter is made up of quotations from Henke and other well-known medico-legal writers. For the present opinions of medical jurists on this subject, we must refer to what has been said in reviewing the work of Jörg (No. XIII. p. 130,) and more recently in the notice of Professor Marc's treatise on Insanity. (No. XIX. p. 170.)

We next have a considerable space occupied by an account of responsibility for illegal acts committed by females while in a state of pregnancy, in which the author appears to follow closely the views of Dr. Jörg. The signs of pregnancy are detailed, in order to show that there is a sufficient want of certainty about them to justify a medical jurist in admitting that, in some cases, females pregnant for the first time may be wholly unconscious of their condition up to the time of parturition. (p. 120.)

In regard to the proofs of abortion, we find nothing more than is commonly stated in most works on midwifery. The subject of responsibility in the parturient state is in great part derived from Jörg's treatise. Dr. Schnitzer, however, differs from that author in regard to his views on the fœtus. Dr. Jörg adopts the eccentric view, that the human fœtus is only a higher species of intestinal worm, that it has not human attributes, and is not therefore a subject for protection by human laws. We have already strongly condemned this doctrine, as unjust and immoral, (B. and F. Med. Rev.,No. XIII. p. 133,) and as one which, if acted upon, would lead to the common practice of abortion.

Dr. Schnitzer censures the views put forth by Jörg, as likely to be subversive of a just administration of the law. As to the objection of that author, that the fœtus does not possess human attributes, because its functions are dormant, and it does not exercise the senses, he observes, the same objection would apply with equal force to a man lying in a state of lethargy, or to one struck by apoplexy or paralysis. If a theory of this kind is to be admitted, it is obvious, as the author remarks (p.370), that infanticide is no longer homicide; and that there can be no greater crime in killing a child than in destroying any domestic animal. Moralists and legislators do not commonly divide murder into different degrees, according to the mental and bodily development of the person killed: but on Dr. Jörg's principle, the killing of a cretin would be a less punishable offence than the killing of a healthy well-developed man. Indeed, to carry this doctrine to its full extent, the crime of murder should always vary in its degree, and therefore in its punishment, according to the intellectual and corporeal development of the party killed!

Dr. Schnitzer in pursuing the subject makes use of much the same arguments as those employed by us in the notice referred to. They are such as would at once occur to most persons who reflected upon the nature and consequences of Dr. Jörg's theory. The sophistical basis on which they rest is too evident to deceive any reader possessed of ordinary acumen. Nevertheless we are not sorry to have met with a German author well fitted to oppose so pernicious a doctrine, emanating from one of his own countrymen, and a physician of no mean reputation.

On the causes of the death of a child during and after delivery, as well as on its "viability" or capability of living, we feel it unnecessary to make any remarks, since these subjects have been recently noticed in this Journal.

The second division of Dr. Schnitzer's book is occupied by an outline of the different forms of insanity. The greater part of this section appears to be compiled from other writers. This circumstance renders it unnecessary for us to occupy our space with quotations. We can find nothing to add to what has already been said on this subject in lately reviewing the works of several modern writers. (B. and F. Med. Rev., No. XIX. July, 1841, p. 129.) We shall only observe, with regard to the treatise before us, that the selections appear to have been made with judgment, and the language is clear and free from mysticism. Those who require merely an abstract of the opinions of modern German authorities on the subject of insanity, will find here what they desire. The author has contrived to weave together a medico-legal description of subjects which are commonly kept quite distinct among English writers; namely, pregnancy, delivery, abortion, infanticide, and insanity; and we must admit that he has shown great skill and ingenuity in blending together such heterogeneous materials.

